# Notice

**Published:** 1874-10

**Authors:** 


### NOTICE.

With pleasure we give place to the following from Dr. Goddard, Treasurer of the American Dental Association.

An opportunity is now offered to those who first apply to procure the Transactions without money and without price.

"At the last meeting of the American Dental Association, held at Detroit, Mich., a resolution was adopted instructing the Treasurer to dispose of the back numbers of their Transactions for the cost of mailing and postage.

I hereby give notice that the Transactions of 1865—66, one volume, can be had by applying to Dr. L. D. Shepard, Hotel Boylston, Boston, Mass., and sending twenty-five cents.

For 1867—70, of Dr. Wm. H. Goddard, Louisville, Ky., the former for fifteen cents the latter for ten cents.

For 1868, of A. M. Leslie and Co., St. Louis, Mo., for fifteen cents.

For 1871—72, one volume, of M. S. Dean, Chicago, Ills., for twenty cents.

For 1873, of Dr. Dean, upon the payment of one dollar.

The Transactions of 1869 were consumed in the great fire in Chicago, Ills.

Persons wishing any of the above will address the parties afore mentioned. None will be sent without the amount specified accompanying the request.

WM. H. GODDARD, Treas.,
65 W. Walnut st., Louisville, Ky.

